# Draft genome datasets for *Cimex hemipterus* from 454 Roche shotgun sequencings and Illumina HiSeq

**DOI:** 10.1016/j.dib.2024.110811

**Published:** 2024-08-09

**Authors:** Li Lim, Abdul Hafiz Ab Majid

**Affiliations:** Household & Structural Urban Entomology Laboratory, School of Biological Sciences, Universiti Sains Malaysia, Penang, 11800 Minden, Malaysia

**Keywords:** Bed bug, Comparison, Assembly, Next generation sequencing, Tropical bed bug

## Abstract

The draft genome data for *Cimex hemipterus* obtained through Illumina HiSeq sequencing were presented. The raw genomic data was deposited in GenBank under BioProject (PRJNA722579) with the BioSample accession number SAMN18780126. Software, including FLASH, SPADES, and QUAST, were used to merge, assemble, and qualify the raw dataset. The assembled genome was available in the Figshare repository. The assembled genomic data was compared to *C. hemipterus* data obtained using 454 Roche shotgun sequencing (BioProject, PRJNA308532), downloaded from NCBI. The draft genome data from this work demonstrated larger data volumes and an updated assembly of the *C. hemipterus* genome with better scaffolding compared to genome data obtained from 454 Roche shotgun sequencing*.*

Specifications Table

**Table:** 

Subject	Genetics
Specific subject area	Genomics and molecular biology
Type of data	Assembled genome data, Table
Data collection	Genomic DNA was extracted from a male tropical bed bug,*Cimex hemipterus*. The genome of tropical bed bugs was sequenced using the Illumina system
Data source location	School of Biological Sciences, Universiti Sains Malaysia, Gelugor, Penang, Malaysia
Data accessibility	The raw genome sequencing data were deposited in NCBI while the assembled data was deposited in Figshare. Both datasets are in FASTQ format Repository name: NCBI, Figshare Data identification number: 1. *BioSample accession number, SAMN18780126 under BioProject (PRJNA722579) 2. Figshare* Direct URL to data: 1. https://www.ncbi.nlm.nih.gov/bioproject/?term=PRJNA722579 2. 10.6084/m9.figshare.16815364
Related research article	None*.*

## 1 Value of the Data

• This genomic sequence data may help to clarify the molecular details of *C. hemipterus* and related traits of this species.
• The genome sequence data of *C. hemipterus* permits researching the genetic information of this species.
• The sequence data will be useful for transcriptome and comparative genomic analyses of *C. hemipterus*.
• To supplement or further enrich the *C. hemipterus* genome sequence data that are currently available.

## 2 Background

Draft genome sequencing of *C. hemipterus*, commonly known as the tropical bed bug, is a critical step in addressing the growing concerns associated with its resurgence and impact on public health. This hematophagous insect has been linked to severe allergic reactions, psychological distress, and the potential transmission of pathogens [1]. Unlike its counterpart, *Cimex lectularius*, the tropical bed bug thrives in warmer climates and has exhibited significant resistance to common insecticides, complicating control measures [2,3]. A comprehensive understanding of its genetic makeup is essential to combat the increasing infestations and develop targeted interventions.

Despite the availability of existing datasets generated using 454 Roche shotgun sequencing, the new dataset is needed as the 454 sequencing technology, though pioneering in its time, has inherent limitations, such as lower throughput, shorter read lengths compared to newer technologies, and higher error rates, particularly in homopolymeric regions [4]. In contrast, Illumina sequencing technology offers significant advancements that can enhance the quality and utility of the genomic data for *C. hemipterus.*

## 3 Data Description

Using an Illumina HiSeq platform, the assembled genome produced a size of 388.66 Mb. The data from the present study was compared with the draft genome data of *C. hemipterus* provided by Seri Masran & Ab Majid, which was downloaded from the NCBI BioProject (PRJNA308532) [5]. In Seri Masran & Ab Majidʼs dataset, the assembled genome is 2.7 Mb [5]. The features of both genome datasets are summarized in Table 1.

**Table 1:** Statistics of assembled sequences of *C. hemipterus*

Measure	Value	
	Present study	Seri Masran & Ab Majid (2016)
System	Illumina HiSeq sequencing	454 Roche shotgun sequencing
Size	388.66 Mb	2.7 Mb
Largest contig (bp)	49,408	575
%GC	35.36	35
N50	8259	519
N75	216	557

## 4 Experimental Design, Materials and Methods

The genomic DNA of *C. hemipterus* was extracted using a HiYield Genomic DNA isolation kit (Real Biotech Corporation, Taiwan) by following the manufacturer's instructions. Before library preparation, gDNA was sheared with Covaris M220 (Covaris, Inc.) to a mean fragment size of around 300 bp. The library was then constructed according to the manufacturer's protocol using the TruSeq™ DNA Sample Prep Kit and cBot Truseq PE Cluster Kit v3-cBot-HS. The draft genome sequence data of *C. hemipterus* was sequenced using the Illumina HiSeq platform (Illumina, San Diego, USA). Generated reads were trimmed using Trimmomatic [6]. The quality of trimmed and filtered paired-end reads was assessed through FastQC [7]. The paired end FASTQ reads were then merged using FLASH (Fast Length Adjustment of SHort reads) [8]. The merged reads were used to identify heterozygosity and genome size estimation through Jellyfish v2.2.10 [9], GenomeScopev1.0.0 [10], and k-mer analysis with the –m 21 option. The reads were then assembled de novo using SPADES genome assembler [11]. The quality of assembled genome was evaluated using QUAST [12]*.*

## Limitations

Not applicable.

## Ethics Statement

Approval from the Human Ethics Commit-tee at Universiti Sains Malaysia (USM/ JEPeM/19,120,868) was obtained.

## CRediT Author Statement

**Lim Li:** Formal analysis; Investigation; Methodology; Project administration; Validation; Writing – original draft; Writing – review & editing. **Abdul Hafiz Ab Majid:** Conceptualization; Funding acquisition; Methodology; Resources; Supervision; Validation; Writing – review & editing.

## Data Availability

Cimex hemipterus Genome sequencing (Original data) (NCBI).
